# Trends in healthcare utilization among older Americans with colorectal cancer: A retrospective database analysis

**DOI:** 10.1186/1472-6963-9-227

**Published:** 2009-12-10

**Authors:** Kathleen Lang, Lisa M Lines, David W Lee, Jonathan R Korn, Craig C Earle, Joseph Menzin

**Affiliations:** 1 Boston Health Economics, Inc, Waltham, MA, USA; 2 GE Healthcare, Waukesha, WI USA; 3 Institute for Clinical Evaluative Sciences, Toronto, ON, Canada

## Abstract

**Background:**

Analyses of utilization trends (cost drivers) allow us to understand changes in colorectal cancer (CRC) costs over time, better predict future costs, identify changes in the use of specific types of care (eg, hospice), and provide inputs for cost-effectiveness models. This retrospective cohort study evaluated healthcare resource use among US Medicare beneficiaries diagnosed with CRC between 1992 and 2002.

**Methods:**

Cohorts included patients aged 66+ newly diagnosed with adenocarcinoma of the colon (n = 52,371) or rectum (n = 18,619) between 1992 and 2002 and matched patients from the general Medicare population, followed until death or December 31, 2005. Demographic and clinical characteristics were evaluated by cancer subsite. Resource use, including the percentage that used each type of resource, number of hospitalizations, and number of hospital and skilled nursing facility days, was evaluated by stage and subsite. The number of office, outpatient, and inpatient visits per person-year was calculated for each cohort, and was described by year of service, subsite, and treatment phase. Hospice use rates in the last year of life were calculated by year of service, stage, and subsite for CRC patients who died of CRC.

**Results:**

CRC patients (mean age: 77.3 years; 44.9% male) used more resources than controls in every category (*P *< .001), with the largest differences seen in hospital days and home health use. Most resource use (except hospice) remained relatively steady over time. The initial phase was the most resource intense in terms of office and outpatient visits. Hospice use among patients who died of CRC increased from 20.0% in 1992 to 70.5% in 2004, and age-related differences appear to have evened out in later years.

**Conclusion:**

Use of hospice care among CRC decedents increased substantially over the study period, while other resource use remained generally steady. Our findings may be useful for understanding CRC cost drivers, tracking trends, and forecasting resource needs for CRC patients in the future.

## Background

Colorectal cancer (CRC) is the third-most common cancer type in the United States (not including basal and squamous cell skin cancer), and the third-leading cause of cancer deaths among both men and women[1]. The total annual cost of CRC care in the US population over age 65 has been estimated at $8 billion in 2002 dollars[2]. To our knowledge, no previous studies have reported comprehensive data on temporal trends in healthcare utilization for CRC patients, although studies have been published on hospice use and end-of-life care among cancer patients (including some CRC patients) [3-11].

In addition to aiding in the understanding of cost drivers, the study of healthcare utilization patterns may lead to cost-saving strategies in the care of CRC (eg, a shift from more-expensive settings to less-expensive settings), and may inform studies regarding the quality of care received by CRC patients as indicated by quality markers including hospice, skilled nursing, and home health care[10]. Inpatient hospitalization is generally the largest cost driver for any disease; therefore, it is vital to be aware of temporal trends in hospitalization use in order to understand changes in costs over time and to predict costs in the future[12]. Finally, decisions on CRC screening can be based on cost-effectiveness analyses and models that rely on CRC cost and utilization studies, and such decisions may depend on having accurate data inputs into these models.

The goals of this study were to analyze the sociodemographic and clinical characteristics of a large cohort of CRC patients, describe healthcare utilization associated with CRC, and assess temporal trends in resource utilization.

## Methods

### Data Source

The data source for this study was the linked Surveillance, Epidemiology, and End Results (SEER)-Medicare database, in which Medicare claims are linked to SEER registry data as part of a collaborative project between the National Cancer Institute and the Centers for Medicare and Medicaid Services. Complete details of the linkage of the SEER and Medicare data have been described elsewhere[13,14]. The study's use of SEER-Medicare data was approved by the National Cancer Institute, assuring patient confidentiality. No other ethics board review was required. In the SEER-Medicare database, patient demographic characteristics (such as age, sex, and race/ethnicity), disease characteristics (such as stage), and resource use details are obtained from hospitals, outpatient clinics, laboratories, private practitioners, nursing homes, hospices, death certificates, autopsy reports, and Medicare claims data[15]. Part A of the Medicare program covers inpatient hospitalizations, skilled nursing facility (SNF) and home health care after a hospital stay, and hospice care. Approximately 99% of Medicare beneficiaries are enrolled in Part A. During this study's analysis period, Medicare did not cover outpatient prescription drugs. Medicare Part B covers physician services (except for routine visits), outpatient services, diagnostic tests, emergency room visits, durable medical equipment (DME), laboratory services, home health care that does not follow a hospital stay, and other medical services and supplies. Approximately 95% of Medicare beneficiaries are enrolled in Part B[13].

### Patient Selection and Follow-up

#### CRC Cohort

All patients aged 66 years and older with a new diagnosis of malignant adenocarcinoma of the colon, rectum, or anus (ie, presence of a SEER cancer site recode value between 15 and 27 and one of the following ICD-O-3 histology codes: 8140, 8210-11, 8220-21, 8260-63, 8470, 8480-81, or 8490) reported to a SEER registry between January 1, 1992 and December 31, 2002 were identified for possible inclusion in the CRC cohort. The index date for each patient was defined as the date of his or her CRC diagnosis.

We excluded patients who were enrolled in a health maintenance organization (HMO) at any point from 12 months pre-index through follow-up. Until recently, HMOs were not required to submit claims for specific services received by their enrollees, so including these patients might have underestimated the total utilization for the sample. We also excluded patients who were not eligible for Part A and B Medicare benefits at any point from 12 months pre-index through follow-up or who were eligible for Medicare benefits based on end-stage renal disease or disability. We excluded patients who had any prior history of cancer, were initially diagnosed with CRC at the time of death or autopsy, were not able to be matched to an appropriate comparator (based on age, sex, and geographic region), or were characterized as having stage 0 or unknown stage disease.

#### Comparison Cohort

Patients in the comparison cohort were randomly selected from a 5% sample of Medicare beneficiaries residing in SEER areas who had not been reported to any of the SEER registries as having CRC. As with CRC patients, comparison patients were excluded if they were enrolled in an HMO or were not eligible for Medicare Part A and B benefits at any point from 12 months before index through follow-up. Comparison cohort patients were not required to have used services in order to be selected for inclusion, and they were allowed to develop cancers other than CRC after their index date. One comparison patient of identical age, sex, and geographic region was matched to each CRC patient and assigned the same index date so that both patients were followed over the same time period. When more than one match was possible, comparators were chosen at random.

#### Follow-up Period

Study patients were followed to evaluate outcomes from their index date until death or the end of the Medicare claims data (December 31, 2005), whichever came first. When a person died before his or her matched comparator, we continued to follow the comparator to record his or her utilization until death or the end of the Medicare claims data, whichever came first.

#### Study Measures

Medicare claims were scanned to identify resource utilization, including physician office visits, outpatient hospital or clinic use, inpatient hospitalization, SNF utilization, and use of home health care and hospice. DME claims were excluded because of incomplete diagnostic coding.

### Data Analyses

The demographic and clinical characteristics of both cohorts were described, including Deyo-Charlson comorbidity scores, which were calculated for the pre-index 12-month period for each person and excluded cancer-related comorbidities[16]. Healthcare utilization was analyzed by cancer subsite (colon or rectal), age at diagnosis, stage at diagnosis, and year of service. The percentages of patients receiving each type of care, number of visits, and lengths of stay were evaluated over the entire follow-up period. We calculated attributable use rates for each phase by subtracting utilization among persons in the comparison cohorts from that among persons in the CRC cohorts over the same time period. This resulted in estimates of the excess numbers of visits per person-year, which were reported by treatment phase for office, outpatient, and inpatient utilization.

Resource use by phase was estimated as follows: 1) terminal-phase resource use was assigned first, with the terminal phase defined as the final year of life (all resource use was considered terminal for patients surviving for less than 13 months after diagnosis); 2) the initial phase was the period, up to 12 months in duration, after diagnosis and before the last year of life among those who lived at least 13 months after diagnosis; and 3) the continuing phase was the period between the first and last year of life among patients with at least 36 months of survival. Resource use by treatment phase was generated by year of service from 1993-2002, the years during which it was possible to fully evaluate each phase.

For our analysis of the use of hospice care, we selected from our overall CRC cohort only those patients who died of CRC during the study period. We then calculated the percentage of patients who used hospice care in their last year of life and reported these data by cancer subsite, year of service, and age at death.

All statistical tests for differences between study and control cohorts were obtained using the Wilcoxon rank-sum test using the Statistical Analysis Software (SAS) package (Version 9.1, SAS Institute, Cary, NC).

## Results

### Patient Demographic and Clinical Characteristics

Selected demographic and clinical characteristics of the CC, RC, combined CRC, and comparison cohorts are shown in Table 1. In the combined CRC and comparison cohorts, the average patient was approximately 77 years of age, 45% were male, and 86% were white. The mean ± SD Deyo-Charlson comorbidity score (excluding cancer) was 0.5 ± 1.0 for cancer patients and 0.4 ± 0.9 for controls; scores were fairly consistent across all years (1992: 0.3 ± 0.8; 2002: 0.5 ± 1.0 in 2002 [data not shown]).

**Table 1:** Demographic and clinical characteristics of patients with CRC and matched controls

	Colon Cancer Cohort		Rectal Cancer Cohort		Overall CRC Cohort		Comparison Cohort	
N	52,371		18,619		70,990		70,990	
Age (mean (± SD)*	77.5	(6.90)	76.5	(6.80)	77.3	(6.90)	77.3	(6.90)
Male*	42.8%		50.5%		44.9%		44.9%	
Race/ethnicity								
White, non-Hispanic	86.2%		86.9%		86.4%		86.8%	
African American, non-Hispanic	7.8%		6.1%		7.3%		6.5%	
Hispanic, any race	1.1%		1.2%		1.1%		1.9%	
Other	5.0%		5.7%		5.2%		4.8%	
Geographic region*								
Northeast	19.3%		19.6%		19.4%		19.4%	
Midwest	27.3%		27.6%		27.4%		27.4%	
West	42.0%		42.4%		42.1%		42.1%	
South	11.3%		10.4%		11.1%		11.1%	
Charlson score (mean (± SD)^1^	0.5	(1.00)	0.4	(0.90)	0.5	(1.00)	0.4	(0.90)
Selected Charlson comorbidities								
Chronic pulmonary/respiratory disease	8.9%		7.9%		8.6%		7.1%	
Congestive heart failure	7.9%		6.0%		7.4%		5.9%	
Diabetes without complications	10.1%		8.4%		9.6%		7.6%	
Cerebrovascular disease	6.0%		4.8%		5.7%		5.7%	
Myocardial infarction	3.2%		2.5%		3.0%		2.6%	
Peptic ulcer	2.4%		1.5%		2.2%		1.2%	
Other major conditions^2^	7.2%		7.2%		7.2%		7.3%	
Stage at diagnosis (%)								
Stage I	23.7%		32.4%		26.0%		**	
Stage II	34.6%		25.1%		32.1%		**	
Stage III	24.6%		22.2%		23.9%		**	
Stage IV	17.2%		20.2%		18.0%		**	

Source: Authors' calculations based on SEER-Medicare data, 1992-2005. ^1^Modified Charlson comorbidity index: excludes cancer-related comorbidities. ^2^Other major conditions include rheumatologic disease, mild liver disease, diabetes with complications, major liver disease, peripheral vascular disease, dementia, renal disease, hemiplegia or paraplegia, and AIDS.

*Variables used in matching cohorts

**Data not available/not applicable

Across all years, CC was most commonly diagnosed at stage II, whereas RC was most commonly diagnosed at stage I. Interestingly, among both CC and RC patients, age at diagnosis changed considerably over the period of analysis. In 1992, 43.7% of CC patients were aged 66-74 years, 40.2% were 75-84, and 16.1% were aged 85 years or more. The corresponding distribution in 2002 was 45.9%, 35.5%, and 18.7%, indicating a trend toward older age at diagnosis (data not shown). In 1992, the age distribution was similar for RC patients: 45.4% were aged 66-74 years, 40.7% were 75-84, and 13.8% were 85 years or older. By 2002, there were more 75-84 year olds (44.3%) diagnosed with RC than 66-74 year olds (40.5%), and the proportion of those aged 85+ years had increased to 15.2% (data not shown).

### Healthcare Utilization

As would be expected, both CC and RC patients used significantly more healthcare resources than matched comparison patients (Table 2). We did not detect a pattern of differences in resource use in the comparison cohort when examined by stage of their matched cancer patient, thus the comparison cohort is shown as a whole. Compared to non-CRC patients, CC and RC patients had an average of about 2 years less follow-up time because of their higher mortality rate. Despite the shorter follow-up, more CRC patients were hospitalized and spent more days in the hospital, more received home health care, and more spent more time in a SNF (all *P *< .001).

**Table 2:** Resource use among patients with CRC, by stage and overall, vs. matched controls

Variables	CRC Patients								Overall CRC Cohort		Comparison Cohort		Difference^1^
						
	Stage I		Stage II		Stage III		Stage IV						
**Colon Cancer Cohort**													
N	12,431		18,095		12,859		8,986		52,371		52,371		
Mean (± SD) months of follow-up	61.1	(39.8)	56.4	(40.9)	44.7	(38.1)	12.7	(19.3)	47.1	(40.7)	68.6	(39.8)	-21.5
Inpatient use	94.4%		97.7%		97.5%		94.3%		96.3%		68.2%		28.1%
Mean (± SD) hospitalizations	3.6	(3.3)	3.6	(3.1)	3.5	(3.0)	2.3	(2.0)	3.4	(3.0)	2.4	(3.1)	1.0
Mean (± SD) hospital days	26.3	(31.8)	28.5	(30.3)	27.9	(26.8)	20.1	(18.9)	26.4	(28.4)	15.1	(25.7)	11.3
SNF use	35.8%		41.2%		38.6%		30.0%		37.4%		29.0%		8.4%
Mean (± SD) SNF days	13.3	(50.9)	15.1	(51.7)	12.5	(37.4)	6.6	(18.4)	12.5	(44.2)	9.7	(33.0)	2.9
Outpatient clinic use	94.5%		91.9%		92.0%		77.4%		90.1%		89.9%		0.1%
Office visit use	99.7%		99.7%		99.6%		98.8%		99.5%		96.2%		3.3%
Home health care use	48.4%		52.9%		53.5%		45.7%		50.7%		36.3%		14.4%
**Rectal Cancer Cohort**													
N	6,041		4,681		4,134		3,763		18,619		18,619		
Mean (± SD) months of follow-up	61.0	(39.9)	52.0	(40.0)	46.0	(37.1)	11.6	(17.3)	45.4	(40.1)	70.6	(40.2)	-25.2
Inpatient use	92.8%		96.8%		98.0%		89.7%		94.3%		67.5%		26.9%
Mean (± SD) hospitalizations	3.6	(3.4)	3.7	(3.0)	3.8	(3.0)	2.0	(2.0)	3.3	(3.0)	2.4	(3.2)	1.0
Mean (± SD) hospital days	26.1	(30.4)	29.5	(28.1)	30.2	(28.0)	16.7	(16.6)	25.9	(27.4)	15.2	(26.0)	10.8
SNF use	35.4%		40.7%		37.3%		30.1%		36.1%		28.1%		8.0%
Mean (± SD) SNF days	13.0	(46.9)	14.2	(40.1)	11.8	(32.8)	6.9	(20.7)	11.8	(38.1)	9.6	(30.3)	2.2
Outpatient clinic use	95.5%		93.4%		95.1%		76.4%		91.1%		90.1%		1.0%
Office visit use	99.6%		99.6%		99.8%		98.8%		99.5%		96.3%		3.1%
Home health care use	54.5%		62.6%		63.9%		46.7%		57.0%		35.3%		21.8%

Source: Authors' calculations based on SEER-Medicare data, 1992-2005. Resource use calculated based on entire follow-up period, expressed on an annual basis.

^1^Difference between overall CRC cohort and comparison cohort. All P < .001.

SNF: Skilled nursing facilities

Note: Cohorts were matched on age, sex, and geographic region

Resource utilization differed somewhat by cancer stage at diagnosis, with stage II and III patients having the most intense absolute service utilization rates by most measures (Table 2). However, when calculated per survival month, stage IV patients had higher utilization rates in all areas. For example, stage IV patients spent more than 2 days per month of follow-up in a hospital or SNF (approximately 1.5 days in a hospital and 0.5 days in a SNF per month), compared to less than 1 day per month of follow-up among patients diagnosed at stages I-III. Use of home health care services ranged between approximately 50-60% for all stages (vs. 35-36% among non-CRC patients), with higher use rates among RC patients.

**Figure 1:**
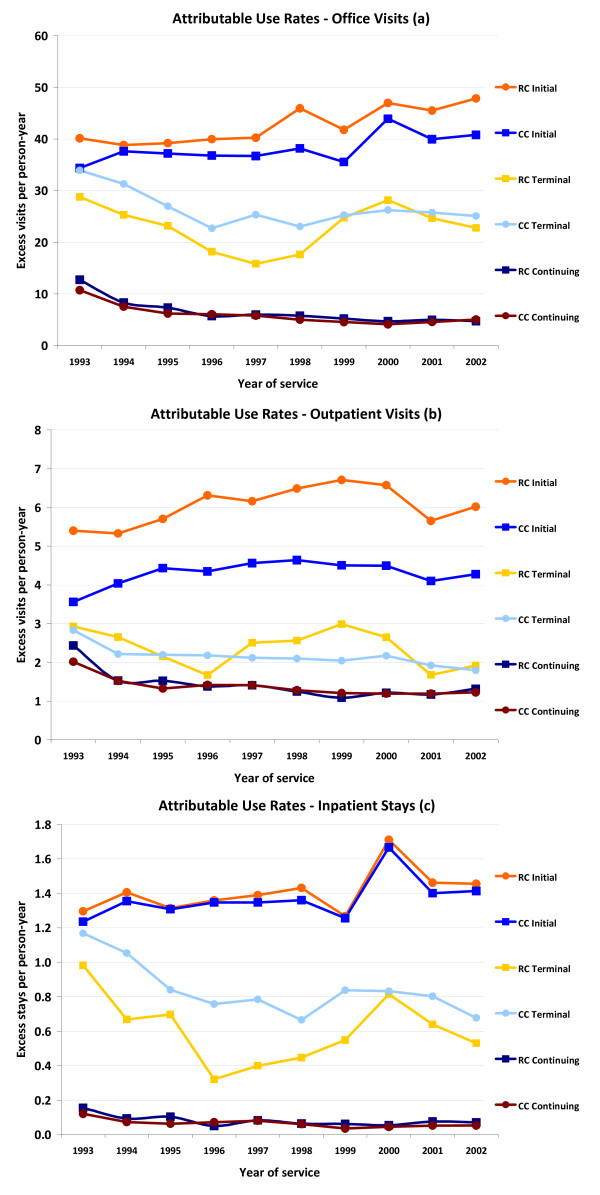
**Excess visits per person-year among patients with CRC, by treatment phase and year of service**.

Figure 1 shows annualized trends in the excess number of office **(a) **and outpatient clinic **(b) **visits and inpatient stays **(c) **by cohort, treatment phase, and year of service (ie, trends in resource use among CRC patients relative to matched comparison patients). Trends in attributable use rates reflect changes among both CRC patients and their comparators. For example, between 1995-1996, office visits decreased by 0.8 visits per person-year among RC patients in the terminal phase and increased by 4.2 visits among comparison patients in the terminal phase, leading to a relative decrease of 5 visits per person year in the number of attributable office visits.

The initial phase of treatment was the most resource intense among both CC and RC patients for all 3 types of resource use, with RC patients having higher excess use rates in most cases in the initial and terminal phases.

**Figure 2:**
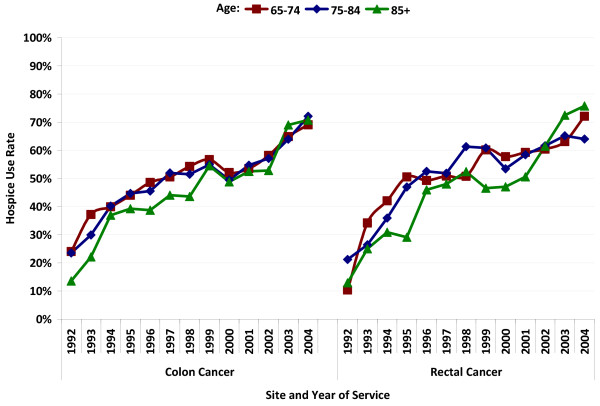
**Hospice use rates among CRC patients who died of CRC, by age at death, year, and subsite**.

Among patients who died of CRC during the study period, an overall proportion of 50.5% of CC and 53.0% of RC patients used hospice services in the last year of life. Among CC patients, the proportion of all decedents who used hospice care in the last year of life increased from 21.6% in 1992 to 71.0% in 2002. Among RC patients, the increase was from 16.1% in 1992 to 69.2% in 2002. In earlier years, persons over age 85 were less likely to use hospice services than younger patients, but this difference seemed to disappear starting around 2002 among both CC and RC patients (Figure 2).

## Discussion

Despite their shorter lifespans, about one-third more CC and RC patients were hospitalized than were matched controls, and CRC patients accrued about 10 more hospital days than did controls. CC patients were hospitalized more often than were RC patients in both inpatient and SNF settings, possibly because of the greater use of surgery in CC patients. RC patients used more home health and hospice services than did CC patients and, in the initial and terminal phases, were more likely to use office, outpatient, and inpatient services. Resource use was most intense among stage IV patients when analyzed per month of follow-up.

During our study period (1992-2005), we observed a marked increase in the percentage of beneficiaries who used hospice care. RC patients had slightly higher hospice use rates than did CC patients, which is interesting in light of the fact that RC patients have lower lifetime and per-lifetime-year costs than do CC patients[17]. The observed increase in hospice use is in line with national trends in hospice use that may have been fueled, in part, by an increase in the number of hospice providers[18]. Medicare spending on hospice services increased by 130% from 2000 to 2004, and the percent of all Medicare beneficiaries who use hospice care increased from 22% in 2000 to 31% in 2004[18]. Given the potential clinical[19] and economic[20] benefits of hospice, it is noteworthy that hospice use increased so dramatically in our sample.

Few previous studies of CRC have included utilization data, and most have focused on hospice use. For example, Lackan et al found an overall hospice use rate of 30.2% among patients with breast, colorectal, lung, and prostate cancer who died between 1991 and 1999[3]. A study by Shugarman et al using 1993-1999 Medicare data to analyze age and gender differences in utilization rates for CRC patients in their last year of life found an overall hospice use rate of 48.0%, with younger patients more likely to use hospice services[11]. As we have shown, it appears that hospice use differences by age may have smoothed somewhat in more recent years.

This study is subject to the limitations of the data source, including potential coding errors, incomplete data, and lack of generalizability to the non-Medicare population [21-23]. While the elderly comprise the majority of patients with CRC, this sample is not representative of all US CRC patients. Despite these limitations, SEER-Medicare data have been used in numerous published studies of CRC[24].

## Conclusion

This retrospective database study of 13 years of data from over 70,000 CRC patients found that Medicare beneficiaries with CRC use significantly more resources than similar individuals without CRC. The most intense outpatient, inpatient, and office visit resource use was seen in the initial treatment phase. Over the study period, the use of hospice among those who died of CRC increased substantially, and age-related differences appear to have moderated over time. Our findings may be useful for understanding changes in costs and cost drivers over time, tracking trends, and forecasting resource needs for CRC patients in the future.

## Competing interests

KL, LML, JRK, JM: Received research funding from GE Healthcare; DWL: Employee of GE Healthcare; CCE: Consultant for Boston Health Economics

## Authors' contributions

KL, JM, DWL, and LML designed the research methods. KL, LML, and JRK collected and analyzed the data. All authors contributed to data interpretation, made substantive contributions to the manuscript, and had final approval of the article.

## Pre-publication history

The pre-publication history for this paper can be accessed here:

http://www.biomedcentral.com/1472-6963/9/227/prepub
